# Pulex Irritans

**Published:** 1892-05

**Authors:** W. A. Yingling

**Affiliations:** Nonchalanta, Kansas


### PULEX-IRRITANS (COMMON FLEA).

W. A. YINGLING, M. D., PH. D., NONCHALANTA, KANSAS.

MIND.—*Very impatient;* can hardly wait to get through with the piece of work in hand; can hardly endure to sit through church service; *restless.*

*Cross and irritable;* does not want to be looked at or spoken to.

*Glad feeling,* as though she had heard good news.

When walking she feels as though she could take ten steps at once a seasily as one from sensation of lightness.

Felt a dread of going to bed; felt like it and did sleep on the lounge.

Memory confused; cannot think.

SENSORIUM.—Sensation of going rapidly, similar to being on a boat sailing rapidly, but no sensation of being on a boat.

Far-away feeling in the head.

*Dizziness* when sitting or standing. (Yet cannot lie down from the vesical trouble.)

Dizziness followed by frontal headache.

INNER HEAD.—Far-away feeling in the head.

Light headed; sense of being unsteady in the head.

Severe frontal headache; better in the open air. When the headache was better the eyeballs were sore, especially on moving them.

Frontal headache in the morning on rising.

Frontal headache better by sitting down quietly.

Frontal headache with which the eyes feel full and large, worse stooping over.

EYES.—*Eyes feel enlarged.*

Both eyes and under part of both lids at edges seem hot and full.

Dark spots before the eyes, closing out part of the vision; spots seem to move with the eyes.

Eyeballs sore after headache.

Eyes feel full and larger with headache.

EARS.—Soreness and suppuration of ringholes in lobes of ears.

External meatus sore and swollen.

Roaring in left ear.

FACE.—Upper lip feels thick; feels like drawing teeth over upper lip.

A careworn expression.

*Wrinkled and an old look.* (Noticed by every one.)

Flushed and very red cheeks.

MOUTH.—Increased flow of saliva.

Peculiar taste, indescribable, but more like steel or some kind of metal.

Perceptible taste of everything eaten at dinner.

Gums bleed easily; tender to the touch.

Severe aching in hollow tooth, extending to face, ear, and jaw; feels as if it would suppurate; swollen.

Breath very foul; can smell and taste it herself; foul like her diarrhœic stool smelled.

Nasty, foul taste in mouth.

Tongue slightly coated white.

A white coating like the skin of an egg gathers on the upper gums, which can be rubbed off with nearly the shape of the gums.

THROAT.—Throat feels very large while swallowing.

Sensation of a hair or thread in the throat.

Throat felt as though it would get sore, but did not.

EATING AND DRINKING.—While eating plums the acid affected the parotid glands (very unusual); the same while eating a not sour apple; glands hurt her.

No appetite.

No thirst at all; water does not taste good.

If she craves anything, which is very unusual, it is something sour or salty.

Aversion to food with nausea and purging.

Drinks little and *not* often with the fever.

No desire for coffee, rather an aversion; formerly craved it.

Desire to drink often, but a little would satisfy.

During the headache very thirsty, could not get enough.

NAUSEA AND VOMITING.—Intense nausea with vomiting, purging, and faintness.

Vomited food she had eaten for supper; slightly sour.

Deathly nausea; "I'm *so* sick."

ABDOMEN.—*Bloating of the abdomen.*

A constant dragging or strain on the muscles of the abdomen.

Sensation that the flatus passed down on the left side of the abdomen to rectum, but seemed then to turn and go upward to the bladder or womb. (She had a peculiar sensation in the vagina like breaking wind, but so indefinite that I did not record it.)

STOOL, ETC.—Pressure in the rectum as from a desire for stool.

An uneasy feeling in the bowels as from a desire for a loose stool.

Diarrhœa very urgent, copious, dark brown, muddy, very foul penetrating odor.

Costive stool in little balls, packed down in rectum with no desire for stool; rectum inactive; accumulation of fœcal matter without desire.

Stool gushing, but in small quantity.

After having diarrhœa for awhile the stool became *very* offensive, the longer the diarrhœa the fouler the stool.

Pressure in the rectum as if it would protrude, with the diarrhœa.

Diarrhœic stool hot and biting.

Stool light yellow and foamy.

Rectum feels drawn and contracted; feels as if full even after relieving bowels by a flushing.

Restless, bowels loose as from taking cold.

URINE.—*Frequent urging* during day; must get up several times at night to pass water.

The flow begins all right, but soon suddenly stops as if closed off, followed with very severe pressure and hurting.

Pressure on the bladder and urethra.

Cannot retain urine, must attend to call at once; wet herself profusely at night before she could reach the vessel; "can't wait a minute."

Bladder very irritable, especially *before* and during menses.

*Urine very scanty with frequent urging;* had to sit on urinal all day with only a few spoonfuls passing during the time; only a few drops at a time.

Urine smells like rotten eggs; dark red.

Burning in the urethra all the time; no better or worse when passing water, but at times the flow comes in a large stream; gushing, which eases the burning. After this free flow of urine the abdomen bloats.

A feeling that a bullet, or something similar, fell in the bladder to its outlet; no feeling remaining after the sense of falling.

Urine milky, with a cloudy sediment, as if mixed with vinegar.

Urine very foul, putrid odor; whitish-red color.

The bladder seems full and large when pressing on the back.

The bladder feels full all the time.

*Tenesmus vesicœ.*

Urine increased, both as to frequency and quantity.

FEMALE SEXUAL ORGANS.—A heavy, pulling sensation below the mammæ.

*Menses delayed;* delayed from three to seven days later at each period; periods getting farther apart.

A full sensation in the veins a week before menses.

Fullness and pressure in the outer parts before flow.

*Blood stains napkin on the edge of stain a deep, muddy green color; stains very hard to wash out.*

Lips dry and blistered before and during the menses; white blisters on lower lip.

Tongue sore and red during menses.

Roof of mouth sore during menses.

Increased flow of saliva during the menses; difficult to prevent its running out of the mouth.

Costive; stool dry, hard, and in balls for a few days before and during menses.

Electric shocks or pulsative shocks at beginning of menses.

Aching in hollow tooth during menses.

External parts are swollen, hot, and tender.

Beating and fluttering in the private parts.

A feeling that she must hold up her parts by the muscles of the abdomen and pelvis; a constant strain on the muscles of the abdomen and parts to hold up.

Feels better sitting up and leaning a little forward, as it seems to support the parts.

Intense burning of the vagina, especially toward the external parts and meatus; burns as if scalding steam were pouring into vagina—continuous without intermission.

Leucorrhœa profuse, yellowish, creamy, very foul, and staining a greenish yellow; extremely hard to wash out.

Discharge of greenish matter, corroding, of a peculiar, nasty smell.

Felt that she must support privates with hands, but too tender to allow her to do so.

Itching of external privates; better scratching.

Sensation as if a small ball of cotton were in the vagina.

For a week before menses feels mean, as if coming unwell; limbs ache; cheeks red and hot; hands and feet cold.

Excerpt from one of the prover's notes: "December 6th, urinary trouble continues; no sediment in vessel on standing. About four o'clock in the afternoon headache and a warm feeling (the glow) goes over me, then I would be chilly for a minute, then warm again. Just before going to bed I became unwell (about nine o'clock); did not flow much during the night; the blood was very light, just colored and that was all.

"December 7th, still very light colored blood; no ache or pain.

"December 8th.—Still no pain or ache; flow darker, most when lying down.

"December 9th.—Flow very dark and stain very hard to wash out. The edges of the stain are very dark-green, almost black; odor very strong; bowels loose.

"December 10th.—Just the same as the 9th.

"December 11th.—A few pains in the forenoon and quit flowing in the afternoon.

"January 22d.—Limbs ache all day; in the evening felt chilly.

"January 23d.—Came unwell in the morning; was sick at the stomach all day, and with dull aching in the lower part of the abdomen. Color very dark, almost black."

Breathing.—Occasionally a sighing breath, broken or hitching, but continuous till it reaches the bottom of the lungs; general relief follows.

Heart and Pulse.—Veins are full to sight, and the blood is perceptibly felt.

Palpitation on going up or down-stairs.

Chest.—Stitches in the left breast.

A heavy pulling sensation below the mammæ.

Neck and Back.—Pain in the back through sacrum.

When she presses on the back the bladder seems full and large.

Drawing of the muscles below scapulæ; feel weak as if strained; better sitting down and at rest.

General weak feeling in the whole of the back.

Backache.

Upper Limbs.—Feels kernels of the axilla on movement as from a cold.

Hands cold.

Lower Limbs.—Feet felt warm and moist as if toasted by the fire.

Aching of the legs between knees and ankles in the bones.

Legs ache from the knees downward.

Constant dull aching, with a tired feeling in the legs.

Some sweating of the feet.

Feels kernels of the groins on movement as from a cold.

Got up with some stiffness of the legs which soon passed off.

Sensation of pushing out on a spot as large as a hand on the inner side of right thigh, without pain.

Feet and ankles very cold.

Dull aching in the thighs.

Feet cold with hot and flushed cheeks; when feet are cold the cheeks are flushed; when feet are warm the cheeks are not flushed.

NERVES.—Very tired feeling all day.

Looks and feels languid and tired, and is dull and cross.

Looks careworn.

Feels as if the flesh and limbs get nervous; can't endure to be at perfect rest.

Electric shocks all through the system, at times start in the body, at others in the head; generally come from motion.

Tired and sleepy; feels as if she had been riding in the wind.

Clothes feel uncomfortable around the body; must remove corset and loosen clothing.

SLEEP.—Wakefulness. Don't feel that she needs sleep.

SKIN.—A prickly itching all over, but more especially on hands and feet, between fingers and toes; rubbing gives temporary relief.

Sore spots all over her, here and there, more on the legs; spots feel bruised to the touch; the spots stay for only a few moments, going and coming rather quickly; spots about one by three inches.

Skin emits foul odor.

Very rapid growth of finger and toe-nails; very thin, almost like isinglass; easily blackened by a bruise.

FEVER.—Feels a heat or glow all over, like being over steam, yet no sweating, but skin is moist.

Feels chilly one day and warm the next.

Flashes of heat over the body.

*Chilliness.* Feels chilly while sitting beside the fire, especially on the back and around the waist; in the evening.

GENERAL.—*Better* by sitting or lying down; moderate warmth; *worse* moving about; cold air; heat.

Feels much better (in fact the only ease secured was) while sitting on the urinal during the vesical trouble and congestion of the female parts.

Sore bruised spots over body and limbs, coming and going rather quickly.

Became very much emaciated; flesh flabby and soft; great weariness.

Looks much older; grows old in looks rapidly; very wrinkled about face and neck.

On going to bed had a warm feeling like heat or a glow for a short time.

Everything worse on the left side with one prover.

RELATIONSHIP.—The delayed menses were restored, and the vesicle trouble relieved by *Nux-vom.*$^{dmm}$ (Swan).

The excessive nausea, vomiting, and purging were relieved by *Ipecac.*$^{dmm}$ (Swan).

The urinary trouble, urging, stoppage in the flow, etc., and the bearing down in the female organs were helped with *Lil-tig.*$^{cm}$ (Swan). At another time with *Puls.*$^{dm}$ (Swan).

The extreme urinary trouble, tenesmus vesicæ, burning in the vagina like steam, swelling of the labia with heat and pressure were relieved with *Sepia*$^{6m}$ (J.).

The toothache was cured like magic with *Nux-vom.*$^{dmm}$ (Swan).

NOTE.—I wish to say that I have not been in search of a new remedy. My wife being so much troubled by the "critters," they being poison to her, raising blotches as severe as a bee sting and as large as a silver half-dollar, I concluded to have Dr. Swan potentize the insect and try to relieve her.

Noticing such marked symptoms I thought a proving might be of value. There surely are some fine effects, and if it proves as effective as a remedy the very great suffering of my wife will not have been in vain.

I trust others will prove the remedy and verify it in practice. Both of my provers used the 1,000th potency. The higher potencies seem to act more promptly, judging from the immediate effect of a dose of the DMM in aggravating all the severer symptoms, especially the vesical and vaginal. This dose was given to see whether the higher would counteract the effect of the lower. Immediately on taking this dose there was a "letting up," relaxation of the strain on the muscles of the abdomen and parts, followed with a very severe aggravation in all symptoms.

I *italize* those symptoms which were present with both provers, and those which were very persistent, coming frequently and severe. All the symptoms recorded were marked and decided; no transient symptoms are given.

To save space I do not fully arrange the symptoms according to the rule, but near enough to be of easy access and intelligence.

